# The spectrum of genetic mutations in myelodysplastic syndrome: Should we update prognostication?

**DOI:** 10.1002/jha2.317

**Published:** 2021-11-01

**Authors:** Michael R. Cook, Judith E. Karp, Catherine Lai

**Affiliations:** ^1^ Division of Hematology and Oncology Lombardi Comprehensive Cancer Center Georgetown University Hospital Washington District of Columbia USA; ^2^ Divison of Hematology and Oncology The Sidney Kimmel Comprehensive Cancer Center Johns Hopkins University Hospital Baltimore Maryland USA

**Keywords:** myelodysplastic syndrome (MDS), oncogenes, prognostic factors, somatic mutation

## Abstract

The natural history of patients with myelodysplastic syndrome (MDS) is dependent upon the presence and magnitude of diverse genetic and molecular aberrations. The International Prognostic Scoring System (IPSS) and revised IPSS (IPSS‐R) are the most widely used classification and prognostic systems; however, somatic mutations are not currently incorporated into these systems, despite evidence of their independent impact on prognosis. Our manuscript reviews prognostic information for TP53, EZH2, DNMT3A, ASXL1, RUNX1, SRSF2, CBL, IDH 1/2, TET2, BCOR, ETV6, GATA2, U2AF1, ZRSR2, RAS, STAG2, and SF3B1. Mutations in TP53, EZH2, ASXL1, DNMT3A, RUNX1, SRSF2, and CBL have extensive evidence for their negative impact on survival, whereas SF3B1 is the lone mutation carrying a favorable prognosis. We use the existing literature to propose the incorporation of somatic mutations into the IPSS‐R. More data are needed to define the broad spectrum of other genetic lesions, as well as the impact of variant allele frequencies, class of mutation, and impact of multiple interactive genomic lesions. We postulate that the incorporation of these data into MDS prognostication systems will not only enhance our therapeutic decision making but lead to targeted treatment in an attempt to improve outcomes in this formidable disease.

## 1 INTRODUCTION

Myelodysplastic syndrome (MDS) is a myeloid neoplasm that occurs predominantly but not exclusively in older adults, with an overall incidence of approximately one to five cases per 100,000, increasing to 20–75 cases per 100,000 in patients aged 65 and above with a median age of 76 [1, 2]. The World Health Organization defines the syndrome based on cytopenias, dysplasia, and certain chromosomal abnormalities [3]. The development of MDS is thought to involve cumulative genetic changes within the hematopoietic stem cell, which can occur over months to years. The natural history of patients with MDS is dependent on the presence and magnitude of diverse genetic and molecular aberrations. The International Prognostic Scoring System (IPSS) and revised IPSS (IPSS‐R) are the most widely used classification systems. In 2012, cytogenetic categories were added to make the IPSS‐R, validated in an independent cohort of 1632 patients [4]; however, molecular mutations are not currently incorporated into any prognostic scoring system.

**FIGURE 1:**
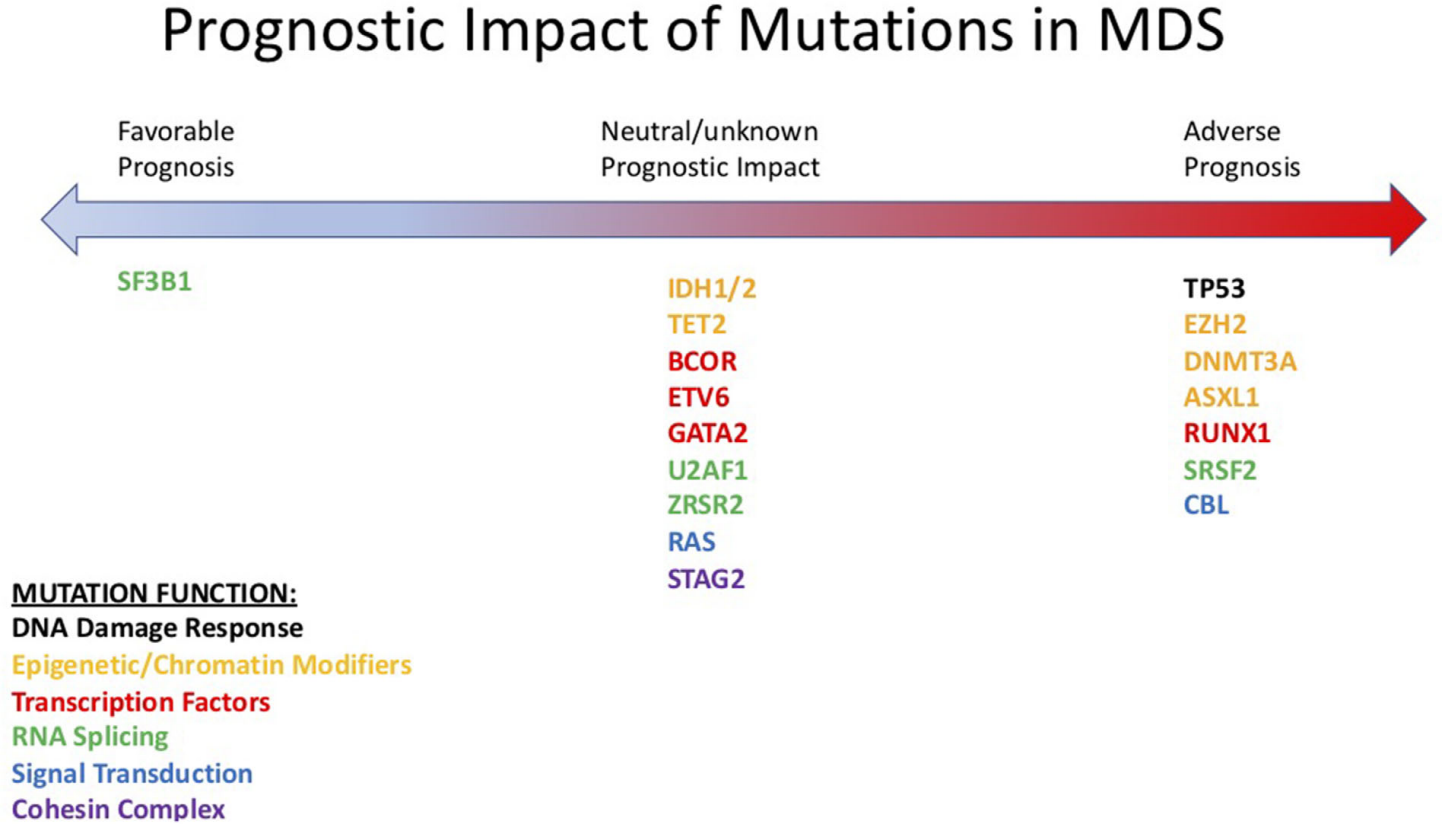
Prognostic impact of mutations in myelodysplastic syndrome (MDS)

Genetic mutations are often detected in patients with MDS with or without other associated structural chromosomal abnormalities. Certain mutations occur preferentially at a higher frequency in genes regulating DNA damage response and apoptosis, epigenetic regulation, transcription factors, RNA‐splicing machinery, activated signaling molecules, and the cohesin complex. In the majority of patients (90%), at least one mutation is present, with a median of three genetic mutations per patient [5]. Many of these mutations are driver mutations and have independent prognostic significance (Figure 1, Table 1). The addition of such molecular information would help to guide discussions of prognosis with patients and aid in determining treatment plans. Moreover, the identification of formative genetic lesions may help to design therapeutic combinations aimed at improving outcomes.

**TABLE 1:** Prognostic information for driver mutations in myelodysplastic syndrome

Oncogenic Mutations	Prevalence	Outcomes:Overall survival (OS)Median survival Event/Progression/Leukemia free survival (EFS, PFS, LFS)
**Mutations associated with favorable prognosis**		
SF3B1	9%–32% [5, 15, 16, 19, 20, 22, 40, 43, 72, 75, 77]	Superior OS (HR 0.10–0.83) [18, 27, 30, 49, 72, 75] Superior median survival (7.5 years) [75] Superior EFS/PFS (HR 0.1–0.29) [72, 75] Superior LFS (HR 0.33–0.44) [30, 75]
**Mutations associated with adverse prognosis**		
TP53	6.3%–21% [5, 16, 19–23]	Inferior OS (HR 1.82‐5.73; RR 9.52) [17–27, 30] Inferior median survival (0.65–0.75 year) [21, 23] Inferior PFS/EFS (HR 3.22–3.97) [19, 21] Inferior LFS (RR 14.66) [22]
EZH2	4.6%–8% [5, 16, 22–24]	Inferior OS (HR 1.55–8.23) [16, 18, 23, 24, 27, 30] Inferior median survival (0.79–0.81 year) [16, 23]
DNMT3A	8%–18% [5, 16, 19, 20, 22, 41, 43]	Inferior OS (HR 1.80‐3.3; RR 1.57) [19, 22, 40, 41] Inferior LFS (HR 2.36–2.7) [40, 41]
ASXL1	10%–31% [5, 15, 16, 19, 20, 22, 23, 43, 47]	Inferior OS (HR 1.21–1.85; RR 1.55) [5, 18, 20, 22, 23, 30, 47–49] Inferior median survival (1.33 year) [23] Inferior LFS (HR 2.17–2.39; RR 2.01) [22, 30, 47]
RUNX1	8%–23% [5, 19, 20, 22, 23, 43]	Inferior OS (HR 1.47–4.59) [5, 18, 20, 23, 24, 48, 49] Inferior median survival (1.16 year) [23]
SRSF2	4%–23% [5, 15, 16, 20, 22, 40, 43, 77, 78]	Inferior OS (HR 1.78–3.3) [40, 77, 78] Inferior LFS (HR 2.83–2.94) [40, 77]
CBL	1.5%–5.1% [5, 23, 24]	Inferior OS (HR 1.57–3.6, RR 2.29) [18, 22, 64]
**Mutations associated with neutral/unknown prognosis** *		
IDH1	0.6%–3.6% [5, 52, 55]	Inferior OS (HR 2.21–4.74) [52–54, 56] Inferior EFS/PFS (HR 2.66) [52] Increased rate AML (67% vs. 28%) [52] Inferior LFS (HR 2.65) [56] Equivalent median OS (22.2 vs. 21.1 months) [57] Neutral OS (HR 1.29, CI: 0.97–1.72) [18]
IDH2	2.1%–4.0% [5, 23, 55]	Inferior OS (HR 1.61; RR 1.95) [22] Equivalent median OS (21.0 vs. 21.1 months) [57] Neutral OS (HR 1.38, CI, 0.95–2.02) [56]
TET2	10%–33% [5, 15, 16, 19, 20, 22, 23, 43]	Inferior OS (HR 2.4) [19] Superior OS (HR 5.2) [59] Superior 5 years OS (76.9% vs. 18.3%) [59] Superior 3 years EFS (89.3% vs. 63.7%) [59] Equivalent median OS (30 vs. 36 months; *p* = 0.37) [60]
BCOR	4.0%–6.1% [5, 24, 64]	Inferior OS (HR 3.3) [64] Equivalent median OS (24.5 vs. 17.9 months, *p* = 0.23) [65]
ETV6	1%–3% [5, 23, 24]	Inferior OS (HR 2.04) [23] Inferior Median survival (0.83 year) [23]
GATA2	0.7%–4.8% [5, 42]	Inferior OS (HR 3.71) [42] Neutral OS (HR 1.19, CI 0.53–2.66) [30]
U2AF1	5%–16% [5, 16, 22, 40, 77, 79]	Inferior OS (HR 1.29) [18] Increased rate AML (15.2% vs. 5.8%) [79] Neutral OS (HR 1.20, CI 0.84–1.72) [16]
ZRSR2	3.1%–11% [5, 22, 24, 40, 77]	Inferior OS (HR 3.3) [40] Inferior LFS (HR 3.6) [40] Neutral OS (HR 0.85, CI 0.65–1.14) [30]
RAS	6.3%‐8.8% [42]	Inferior OS (HR 1.83–3.52) [30, 42] Neutral OS (HR 1.60, CI 0.76–3.35) [16]
STAG2	5.9%–7.5% [5, 22, 24, 84]	Inferior OS (HR 1.5–1.5) [30, 84]

*Note*: Prognostic data for MDS driver mutations, including strength and statistical significance of prognosis via hazard ratios, relative risk, and median OS. Table divided into mutations associated with favorable prognosis, adverse prognosis, and unknown prognosis. Overall Survival (OS): hazard ratio's (HR) time of sample collection/time of diagnosis to the time of death from any cause or last follow up. Progression free survival (PFS): time of transplantation to date of relapse, progression, or death. Leukemia free survival (LFS): time of sample/diagnosis to confirm Leukemic transformation. Event‐free survival (EFS): defined as progression to AML or death.

* Some mutations show only inferior outcomes but are listed under neutral/unknown prognosis because data comes from small studies or lack of clear evidence that mutation is associated with adverse outcome.

## 2 MUTATIONS AFFECTING DNA DAMAGE RESPONSE

### 2.1 TP53 mutation

TP53 tumor suppressor gene provides instruction for creation of tumor protein p53, which acts as a tumor suppressor by mediating cell cycle arrest in response to a variety of cellular stressors [6]. TP53 mutations at the time of diagnosis are found in 6%–21% of MDS patients and are more frequently associated with complex chromosomal abnormalities, and prior exposure to alkylating agents or radiation (therapy‐related MDS) [7, 8, 9].

Complex cytogenetics (more than three chromosomal abnormalities) are frequently associated with TP53 mutations [10]. However, TP53 mutations were found to be independently worse than TP53_wt_ with complex cytogenetics, showing an inferior overall survival (OS) and median survival time [10, 11]. Furthermore, patients with TP53 mutations, independent of complex cytogenetics, have poor outcomes in terms of OS, median survival, progression‐free survival (PFS), leukemia‐free survival (LFS), and survival following an allogeneic hematopoietic stem cell transplant (HSCT). These findings are independent of age, prognostic scoring system, blast count, hemoglobin, or other genetic mutations [5, 12–30]. Despite favorable prognosis associated with an isolated del(5q), the presence of TP53_mut_ confers a markedly lower median survival (4.8 months vs. 48.8 months in low‐risk MDS) and increased 5‐year risk of acute myeloid leukemia (AML) compared with wild type [31].

Equally important, there appears to be significant heterogeneity within TP53 mutations with respect to variant allele frequency (VAF) and number and class of TP53 mutation affecting survival. Higher VAFs (20%–50% and >50%) were associated with a worse prognosis, whereas VAF < 10% did not have a statistically different survival when compared to TP53_wt_ without complex cytogenetics [32]. Moreover, TP53 aberrations can present as a mutation, deletion, or copy‐neutral loss of heterozygosity (cn‐LOH). Patients whose MDS cells harbor a combination of mutation and deletion/cn‐LOH constitute so‐called biallelic or multi‐hit TP53 disease. Interestingly, patients with biallelic TP53 lesions or losses have more severe cytopenias, higher bone marrow blast percentages, and shortened median OS (8.7 months vs. 42 months) relative to monoallelic TP53 mutation without deletion/cn‐LOH in the other allele [33].

## 3 MUTATIONS IN EPIGENETIC REGULATION OF GENE EXPRESSION

### 3.1 EZH2 mutation

Enhancer of zeste homolog 2 (EZH2) is a histone methyltransferase, forming part of a protein called polycomb repressive complex‐2, and is involved in cell fate determination of immature forms [34]. These mutations occur in 4%–8% of patients with MDS, and have been associated with chromosome seven abnormalities [35], lower median platelet counts (68,000 vs. 112,000), and a trend toward higher IPSS‐R score and bone marrow blast percentages [36]. Patients harboring this mutation are noted to have shortened survival when controlling for blast count, hemoglobin, other genetic mutations, prognostic scoring systems, and age [16, 18, 23, 24, 27, 30, 36]. Direct comparisons to EZH2_wt_ patients show those lacking this mutation appear to live longer [5, 16, 35, 36]. To illustrate these poor outcomes, a combined sampling of 2243 patients showed EZH2 mutations were associated with inferior OS (hazard ratio of 2.37, CI 1.49–3.79) [37].

### 3.2 DNMT3A mutation

This gene encodes DNA methyltransferase‐3‐alpha, which is involved in DNA methylation, promoting hematopoietic stem cell differentiation into progenitor cells [38]. DNMT3A mutations are among the most common, found in 8%–18% of cases. Patients are older (74 vs. 66 years) with higher platelet counts (123,500 vs. 73,000) at diagnosis. Furthermore, 48% of patients with DNMT3A mutations carry additional genetic co‐mutations, in particular IDH1/2 mutations [39]. When controlling for age, gender, IPSS‐R, and other mutations, DNMT3A_mut_ patients are more likely to undergo leukemic transformation and succumb to their disease [19, 22, 40–42]. DNMT3A_mut_ has lower median OS (15 vs. 32 months) and higher rates of AML transformation at 5 years compared with DNMT3A_wt_ [40, 41, 43, 44]. Consequently, poor outcomes were noted in 12 studies spanning 2236 patients with cumulative shortened survival (hazard ratio 1.65, *p* < 0.001) and survival free of acute leukemic transformation (hazard ratio 4.62, *p* < 0.001) 45].

### 3.3 ASXL1 mutation

An epigenetic regulator, additional sex‐comb like‐1′ gene, is frequently overexpressed in myeloid malignancies. This overexpression in mouse models impairs myeloid differentiation and induces MDS [46]. ASXL1 mutation occurs in 10%–31% of patients with MDS and is associated with poor clinical outcomes. Factoring in patient age, gender, IPSS, MD‐Anderson Lower‐Risk Prognostic Scoring System, another mutational status, and karyotype, ASXL1 mutations confer decreased OS, inferior LFS, and increased rate of relapse following allogeneic HSCT [5, 18, 20, 22, 23, 30, 47–49]. Additionally, MDS without ASXL1 mutation exhibits improved survival and less progression to AML [15, 16, 43, 47]. Further illustration of ASXL1 impact is with 20q deletion. In MDS, where a 20q deletion is considered to have a favorable prognosis, combination with ASXL1 overexpression confers an inferior 2‐year OS (45.5% vs. 87.9%) [50].

### 3.4 IDH mutations

There are two isocitrate dehydrogenase genes, IDH1 and IDH2, both of which produce enzymes that convert isocitrate to 2‐ketoglutarate to produce cellular energy. Mutations in these genes are common in several cancers, resulting in overproduction of (R)‐enantiomer of 2‐hydroxyglutarate, which is thought to promote leukemogenesis [51]. In general, these are uncommon mutations in patients with MDS; IDH2 mutations occur in 2.1%–4.0% compared with IDH1 mutations in 0.6%–3.6% of patients.

It is unclear how this mutation affects patient outcomes. IDH1 mutations have been linked with inferior LFS and OS when controlling for factors such as karyotype, transfusion dependence, and IPSS [52, 53, 54]. While IDH2_mut_ compared with IDH2_wt_ confers inferior survival and increased probability of relapse following HSCT, poor survival and increased rate of relapse associations for IDH2_mut_ remained statistically significant in only one of five datasets [15, 18, 20, 22, 55]. Strengthening these findings is a meta‐analysis of 1782 MDS patients across six studies that detected IDH mutations in 111 patients (6.2%), which predicted poor prognosis, with inferior OS (HR 1.62; CI, 1.27–2.09) and LFS (HR 2.21; CI, 1.48–3.30). Inferior OS appeared to be driven predominantly by IDH1 mutations (HR 2.21; CI, 1.53–4.5), whereas there was a marginal, not statistically significant, inferior OS for IDH2 mutations (HR 1.38, CI, 0.95–2.02) [56]. Conversely, a separate large dataset of 1042 MDS patients found no difference in overall survival for either mutation compared to the wild‐type population (OS: IDH1 22.2 months, IDH2 21.0 months, IDH_wt_ 21.1 months). There was also no difference between the groups when comparing complete response rates to hypomethylating agents [57].

### 3.5 TET2 mutation

The Ten‐Eleven translocation gene is a probable tumor suppressor gene altered in hematologic malignancies and may have key functions in normal hematopoiesis and hematopoietic stem cell biology [58]. While TET2 mutations occur relatively frequently in MDS, detected in 11%–33% of cases, the impact of these mutations on prognosis is unclear. TET2 mutations have been independently associated with a shortened survival following allogeneic HSCT [19]. In contrast, TET2 mutation was found to have improved 5‐year OS (76.9% vs. 18.3%), 3‐year LFS (89.3% vs. 63.7%), and 4.1‐fold decreased risk of death when compared to TET2_wt_ controlling for age, IPSS, and transfusion requirement [59]. Furthermore, survival and transformation to AML did not relate to TET mutational status in a 355‐patient dataset, as well as in a pooled 1494 patient analysis [60, 61]. These inconsistent results may be explained by VAF of TET2 mutations; shortened survival (20.4 vs. 47.8 months) was noted for TET2_mut_ with VAF > 18% when compared with composite of VAF < 18% or TET2_wt_ [62].

## 4 TRANSCRIPTION FACTOR MUTATIONS

### 4.1 BCOR mutation

BCOR is a repressor of the HoxA gene cluster in myeloid cells, and loss of BCOR is hypothesized to provide a clonal growth advantage to MDS cells [63]. It is found in approximately 4%–6% of patients and is commonly associated with RUNX1 and DNMT3A mutations. Patients with BCOR mutations may have an inferior OS when controlling for age, IPSS, transfusion dependency, and mutational status [15, 43, 64]. Conversely, a study of 621 patients showed BCOR_mut_ status detected no difference in median OS (24.5 vs. 17.9 months, *p* = 0.23) compared with BCOR_wt_, or after adjustment for age and IPSS‐R risk categories. Nonetheless, the type of mutation may be an important determinant, as patients with frameshift BCOR mutations were found to only have a median survival of 10 months, compared with 50 months for all other types of BCOR mutations (missense, stop‐gain, nonsense) [65].

### 4.2 RUNX1

This gene produces runt‐related transcription factor 1 that interacts with core‐binding factor beta and binds DNA to prevent it from being degraded. RUNX1 protein activates genes to control hematopoietic stem cells [66]. RUNX1 mutations occur in 8%–23% of MDS, most commonly in the setting of therapy‐related MDS, and are frequently detected in patients who transform to AML [67]. Given these associations, it is not surprising that outcomes in this patient population are quite grim. Controlling for factors such as MD Anderson Prognostic Scoring system, IPSS/IPSS‐R, other genetic mutations, blast count, and hemoglobin levels, multiple studies have found that patients whose cells harbor RUNX1 mutations have shortened survival and increase the rate of relapse following allogeneic HSCT [5, 18, 20, 23, 24, 48, 49]. Moreover, patients lacking this mutation have been shown to live longer and have a longer time to acute leukemic transformation [5, 15, 16, 18, 20, 24, 43, 68].

### 4.3 Other transcription factors

The ETV6 gene encodes a hematopoietic transcription factor that functions in early hematopoiesis in the bone marrow [69]. It is an uncommon mutation in MDS, occurring at a rate of 1%–3%. ETV6 mutations have been correlated with poor outcomes, showing decreased median OS (0.83 years) and inferior OS when controlling for other prognostic factors [23]; however, this evidence has not been described elsewhere in the literature.

GATA1/2 are protein transcription factors, which regulate the growth and proliferation of immature red blood cells and megakaryocytes. Inherited and acquired mutations in GATA1 have been shown to lead to abnormal and malignant hematopoiesis [70], and GATA2 mutations have been associated with mononuclear cytopenias, MDS, and AML [71]. Prognostic data appear immature. Survival was found to be inferior for GATA1/2_mut_ MDS when controlling for age, cell counts, mutational status, and IPSS‐R [42]. Alternatively, this survival disadvantage was lost in a separate dataset for when controlling for similar prognostic factors [5].

## 5 MUTATIONS IN RNA SPLICING

Next‐generation sequencing approaches have identified mutations in multiple RNA splicing genes. Collectively, these mutations have been observed in 85% of neoplasms with MDS features, and approximately half of MDS patients carry at least one somatic mutation in a spliceosome gene [72, 73, 74]. Those mutations likely affect the core components of initial steps in the RNA splicing machinery, but the link to leukemogenesis remains elusive [40].

### 5.1 SF3B1 mutation

This gene encodes for the splicing factor 3b subunit 1, and it occurs in approximately 25% of patients. Based on a recent update from the international working group of experts in MDS, SF3B1‐mutant MDS now refers to a new subtype of the disease. It is defined by the mutation, cytopenias, morphologic dysplasia (with or without ringed sideroblasts), bone marrow blasts < 5%, and peripheral blasts < 1%. This distinction was made as SF3B1_mut_ is classically characterized by a ringed sideroblastic phenotype with ineffective erythropoiesis but an indolent clinical course [74]. Undoubtedly, patients whose cells harbor this genetic alteration live longer (7.5 vs. 4.1 years) with less progression to AML, independent of age, IPSS‐R, or other somatic mutations [18, 27, 30, 72, 75]. Further confirming the association, SF3B1_mut_ compared to SF3B1_wt_ found this mutation to possess a superior OS [5, 15, 30].

### 5.2 SRSF2 mutation

This gene encodes serine/arginine‐rich splicing factor 2 that belongs to the serine/arginine rich protein family, which is important for splice‐site selection, splicesome assembly, and both constitutive and alternative splicing [76]. This subtype occurs in 4%–23% of patients and is associated with excessive blasts, mild thrombocytopenia (88,000 vs. 165,000), and decreased neutrophils (1100 vs. 2300) [40]. Unlike SF3B1_mut_ patients, SRSF2_mut_ have relatively poor disease outcomes with increased rate of leukemic progression and inferior survival when controlling for age, IPSS risk groups, transfusion dependence, and other gene mutations [40, 77]. Furthermore, when compared with wild type, this mutation is linked to inferior OS and progression to AML [15, 16, 18, 40, 77]. Finally, a grouped analysis of 2056 patients across 12 studies predicted inferior survival (pooled HR's for MDS patients = 1.780, CI 1.410–2.249) for this subtype [78].

### 5.3 U2AF1/ZRSR2 mutation

These genes (U2AF1‐U2 small nuclear RNA auxiliary factor 1; ZRSR2‐U2 small nuclear ribonucleoprotein auxiliary factor 35 kDa subunit‐related protein 2) are involved in the spliceosome pathway and are reported to be mutated in approximately 5%–16% and 3%–11% of MDS cases, respectively. Both mutations have been linked to inferior outcomes, however in comparison to other genetic lesions the amount of evidence is small. U2AF1_mut_ patients have been linked to inferior survival when controlling for IPSS‐R/other genetic mutations [18], and increased rate of leukemic transformation when compared to wild‐type patients [79]. Conversely, inferior survival noted in U2AF1_mut_ compared to U2AF1_wt_ disease did not meet statistical significance when considering other prognostic variables [16]. Similarly, ZRSR2 mutations have been linked to inferior OS and increased rate of AML transformation [40], but this observation is unconfirmed to date.

## 6 MUTATIONS INVOLVED IN SIGNAL TRANSDUCTION

### 6.1 CBL mutation

The Casitas B‐lineage (CBL) lymphoma gene encodes a tumor suppressor protein with E3 ubiquitin ligase activity that acts as a negative regulator of receptor tyrosine kinase. As such, a lack of this signal drives an oncogenic pathway [80]. Mutant CBL is thought to play an important role in development of myeloid malignancies [81], and in MDS the mutation occurs in 1.5%–5.1% of cases. Regardless of its incidence, CBL mutant patients have poor outcomes compared with wild‐type CBL. Importantly, independent inferior survival was noted despite controlling for other prognostic markers such as age, transfusion dependence, IPSS, and other mutations [18, 22, 64].

### 6.2 RAS pathway mutations

This group includes NRAS, KRAS and HRAS that encode GTPases that regulate cell proliferation, differentiation, and survival [82]. While mutations in the RAS gene family are found at a rate of 6.2%–8.8% of MDS, there does not appear to be definitive relation to prognosis. Multiple separate analyses show lack of a prognostic impact when controlling for age, cell counts, IPSS‐R, and other mutations [5, 16, 29]. Alternatively, two studies have associated these mutations with inferior OS when controlling for established clinical risk factors [30, 42]. Other RAS pathway mutations, PTPN11 and JAK2, are uncommon in MDS, but also part of this signal transduction pathway. No independent survival impact was found for PTPN11 or JAK2 in similar analyses [5, 29].

## 7 COHESIN COMPLEX MUTATIONS

The cohesin complex includes a multitude of genes that act together in the control of cell division through regulating dissociation of sister chromatids in mitosis or meiosis [83]. Mutations that occur within this complex in MDS are SMC1A, RAD21, SMC3, and STAG2. STAG2_mut_ MDS appears to be the most frequently mutated gene in the complex, occurring in 5.9%–7.5% of cases. Collectively, the impact of cohesin complex mutations on prognosis has yet to be defined. STAG2_mut_ disease has been associated with inferior survival independent of age, gender, and IPSS [30, 84]. Despite this finding other data suggest the association with poor survival is lost when controlling for known prognostic markers, even when considering cumulative mutations in the entire cohesin complex (STAG2, SMC3, RAD21/ SMC1A) [5, 18, 24]. In a large analysis, RAD21 mutations were found to predict inferior survival when controlling for age and IPSS‐R, however this has not been duplicated elsewhere in the literature [30].

## 8 CLINICAL APPLICATION OF GENETIC MUTATIONS

**FIGURE 2:**
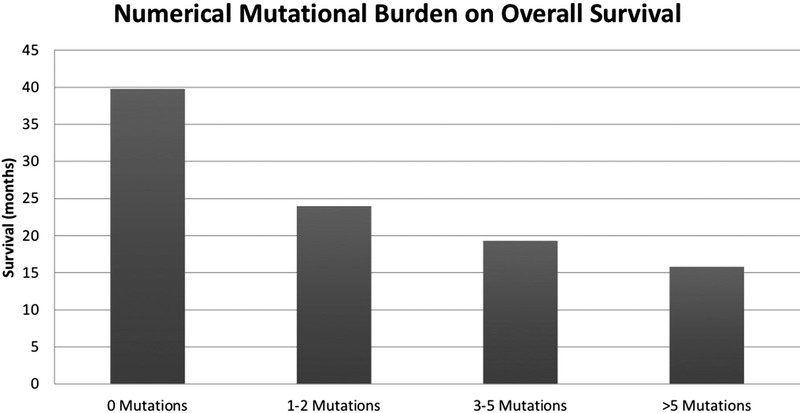
Numerical mutational burden and impact on overall survival (adapted from Nazha et al. [43]). Graphical representation of mutational burden and overall survival (listed in months) for any genetic mutations found by evaluating coding exons of 29 genes using the Illumina TruSeq custom amplicon kit and sequenced on the Miseq, San Diego, CA, USA

Importantly, both the specific mutational profile and the overall mutational burden influence OS (Figure 2; mutational profiles prepared for coding exons of 29 genes using the Illumina TruSeq Custom Amplicon kit and sequenced on the Miseq, San Diego, CA, USA). MDS patients with zero mutations had improved OS compared to those with one to two mutations, three to five mutations, and more than five mutations, respectively (median OS 39.8 vs. 24 vs. 19.3 vs. 15.8 months) [43]. This exponential decline in prognosis for increased mutational burden has been corroborated across various endpoints. Patients with one mutation had a 20% reduced median survival at 40 months (90% vs. 70%) compared to those without molecular abnormalities [24]. In addition, the rate of leukemic transformation (median rate 4 months with ≥5 mutations) and time spent free of disease progression (HR 3.364 for PFS) were inferior for those with higher mutational burden [15, 17, 24, 42].

Recent data suggest that driver mutations emerge heterogeneously in different patient subgroups. VAF, or the percentage of a specific genetic variant, is gaining traction for mutational prognostication [85]. For example, in the case of TP53, patients with VAF < 10% are not found to have the same poor outcomes as those with higher VAFs [32]. In contrast, patients who harbor TET2 mutations with a VAF > 18% have been shown to have a 57% reduction in lifespan, when compared to patients with VAF < 18% or TET2 wild‐type disease. Additionally, SF3B1 mutations with a VAF > 15% were not only seen to have superior survival, but more likely to be classified as MDS with ringed sideroblasts [62].

In 2012, the IPSS was updated to include cytogenetics [86]. Using comparable OS and leukemic free survival data, we postulate a scoring system (Table 2) that would be able to capture the survival loss or gain described in the literature for TP53, EZH2, ASXL1, DNMT3A, RUNX1, SRSF2, CBL, SF3B1, and the cumulative impact of patients with multiple molecular abnormalities. Values in Table 2 were assigned to genetic mutations based on comparable median survival or hazard ratios for overall survival/progression to AML for cytogenetic abnormalities. For example, SF3B1 has been noted to have a median OS of 7.5 years, outperforming the 5.4 year median OS for “Very Good” cytogenetic abnormalities [−Y, del[11q]] [87]. Full comparison of outcome measures is found in Tables 3 and 4 [4, 86, 87].

**TABLE 2:** Proposed update to the IPSS‐R

Prognostic variable					Score			
	−1.0	0	0.5	1.0	1.5	2.0	3.0	4.0
Genetic mutation	SF3B1					DNMT3A, ASXL1, RUNX1, SRSF2, CBL	EZH2	TP53
Number of genetic mutations		0				1−2	≥3	
Cytogenetics		Very good		Good		Intermediate	Poor	Very poor
Bone marrow blast (%)		<2		>2 – < 5		5–10	>10	
Hemoglobin		≥10		8 – < 10	<8			
Platelets		≥100	50–100	<50				
Absolute Neutrophil Count		≥0.8	<0.8					

**TABLE 3:** MDS cytogenetic scoring system, modified from Greenberg et al. [86]

Prognostic subgroup	Cytogenetic abnormality	Median survival, year^a^	Hazard ratios OS/AML^a^	Hazard ratios OS/AML^b^
Very good	−Y, del(11q)	5.4	0.7/0.4	0.5/0.5
Good	Normal, del(5q), del(20q), del(12p) double including del(5q)	4.8	1/1	1/1
Intermediate	del(7q), +8, +19, i(17q), any other single or double independent clones	2.7	1.5/1.8	1.6/2.2
Poor	–7, inv(3)/t(3q)/del(3q), double including –7/del(7q), complex: 3 abnormalities	1.5	2.3/2.3	2.6/3.4
Very poor	Complex: >3 abnormalities	0.7	3.8/3.6	4.2/4.9

^a^ Data from IWG‐PM database (*n* = 7012).

^b^ Data from Schanz et al. (*n* = 2754). Proposed change incorporates mutations that hold inferior and superior prognostic information, as well as total burden of mutations.

**TABLE 4:** Genetic mutation scoring system

Prognostic subgroup	Molecular abnormality	Median survival, year	Hazard ratios, OS	Hazard ratios, AML
Favorable	SF3B1	7.5	0.10–0.83	0.33–0.44
Very good				
Good				
Intermediate	DNMT3A ASXL1 RUNX1 SRSF2 CBL	1.25 1.33 1.16	1.80–3.30 1.21–1.85 1.47–4.59 1.78–3.30 1.57–3.60	2.36–2.7 2.17–2.39 2.43 2.83–2.94
Poor	EZH2	0.79–0.81	1.55–8.23	
Very poor	TP53	0.65–0.75	1.82–5.73	14.66

Tables 2‐4 detail a proposed change to the IPSS‐R, by comparing median survival, hazard ratios for overall survival, and transformation to AML, to similar cytogenetic data that was used to create the initial IPSS‐R model in 2012.

Please refer to Table 1 for full list of citations regarding overall survival and leukemic free survival for each individual mutation.

A similar algorithm was recently created from 1471 MDS patients, incorporating the standard prognostic variables (karyotype, platelet count, hemoglobin, bone marrow blast percentage, age) and seven discrete genetic mutations (TP53, STAG2, RUNX1, RAD21, SRSF2, ASXL1, and SF3B1) that had independent impact on overall and leukemic free survival [30]. Other changes to the standard prognostication models include streamlined whole‐genome sequencing of patients. A study of patients with AML and MDS using whole‐genome sequencing detected all standard genetic alterations by traditional cytogenetic analysis, as well as identify a new genomic event in 25% of this population, leading to a change in prognostic risk category for 16.2% of the cohort [88]. A separate analysis of older (≥60) AML patients was able to show dramatically different outcomes for patients treated with traditional 7 + 3 induction chemotherapy, when the mutational status of seven genes (NPM1, ASXL1, DNMT3A, FLT3‐ITD, NRAS, TP53, KRAS) was added to traditional cytogenetics to create the ALFA decision model, which was validated among a three large (*n* = 830) cohorts [89]. While this model was used in AML patients one can suggest that a similar algorithm could be applied to MDS, as high‐risk MDS patients are often treated similar to those with AML. Whether an addition to the IPSS‐R, or an entirely new model similar to that suggested by Nazha and colleagues, we feel molecular data need to be widely implemented in prognostication systems.

## 9 GENOMIC‐BASED THERAPEUTICS

Understanding genetic drivers of MDS provides opportunity to target these lesions (Table 5) and improve patient outcomes. Signaling via the SMAD2‐SMAD3 pathway is constitutively increased in patients with MDS, yielding an inhibitory effect on red cell maturation [90]. Luspatercept, a recombinant fusion protein that binds transforming growth factor β superfamily ligands to reduce SMAD2/3 signaling, is now FDA approved for the treatment of very low to intermediate risk MDS with ring sideroblasts and SF3B1 mutation. This approval is based on a recent phase III study showing transfusion independence for 38% of the Luspatercept arm, compared to 13% of the placebo group (*p* < 0.001) [91].

**TABLE 5:** Targeted therapeutics currently under investigation

Genetic mutation	Therapeutic drug	Clinical trial number	Phase
TP53	Azacitidine + APR‐246 Magrolimab + azacitidine Azacitidine + APR‐548	NCT03745716^b^ NCT04313881^c^ NCT04638309^c^	III III I
ASXL1	FT‐1101 + azacitidine RO6870810 CPI‐0610 +/– ruxolitinib	NCT02543879^b^ NCT02308761^b^ NCT02158858^c^	I/Ib I I
IDH1	FT‐2102 +/– azacitidine or cytarabine AG‐120 Ivosidenib/venetoclax +/– azacitidine IDH305 Ivosidenib + cytarabine/fludarabine	NCT02719574^c^ NCT03503409^c^, NCT02074839^c^ NCT03471260^c^ NCT02381886^a^ NCT04250051^c^	I/II II I I/II I I
IDH2	AG‐221	NCT03744390^c^ NCT01915498^a^	II I/II
IDH1/2	Ivosidenib (IDH1) or enasidenib (IDH2) + induction therapy vs. placebo + induction AG‐881 Olaparib	NCT03839771^c^ NCT02492737^b^ NCT03953898^c^	III I II
RNA splicing machinery	GSK3326595 H3B‐8800	NCT03614728^c^ NCT02841540^a^	I/II I
Signal transduction	Azacitidine + quizartinib LGH447 vs LHG447 + midostaurin Rigosertib Azacitadine, venetoclax, trametinib Pevonedistat + azacitidine	NCT04493138^c^ NCT02078609^b^ NCT02562443^b^ NCT04487106^c^ NCT03268954^c^	I/II I III II III
Cohesin complex	Talazoparib	NCT03974217^c^	I

^a^ Active, not yet recruiting.

^b^ Trial completed or temporarily suspended.

^c^ Active and recruiting.

In TP53 mutant disease, APR‐246 (eprenetapopt), a novel drug that promotes refolding of TP53 and restoration of function, initially had encouraging results in a phase Ib/II clinical trial in combination with azacitidine (at 10.5 months, 45 patients with AML or MDS overall response rate [ORR] was 87%, CR rate of 61%) [92]. Unfortunately, the phase III trial of eprenetapopt + azacitidine compared to azacitidine alone failed to meet the prespecified primary endpoint of improved CR, although final results are still pending. Numerically the CR was improved for the combination (33.3% vs. 22.4%), but failed to meet statistical significance [93]. APR‐548, a second generation p53 activator, is currently under phase I investigation in combination with azacitidine, however there are no preliminary results at this time. Magrolimab, an antibody blocking the CD47 macrophage immune checkpoint, induces phagocytosis and destruction of leukemic stem cells. Preliminary data presented at the 2020 American Society of Clinical Oncology conference showed a 91% ORR and 42% CR rate in 33 MDS patients at time of data cutoff. Importantly, responses were also seen in TP53 mutant AML patients, necessitating further study in both TP53_wt_ and TP53_mut_ MDS [94].

IDH1/2 inhibitors are now approved in AML and are being studied in MDS. A phase I study of IDH1 inhibitor FT‐2102 in 28 patients with AML or MDS showed an ORR of 32%. However, only four patients had MDS, one of whom had a CR [95]. A similar phase I study of FT‐2102 in combination with azacitidine had comparable efficacy with an ORR of 42% in 24 patients with relapsed/refractory (R/R) AML or MDS, but again, only one MDS patient was included in this analysis [96]. A second study of 12 patients with R/R MDS who received oral ivosidenib, five patients achieved CR and three of those remained relapse free at 12 months [97].

There are multiple therapeutics currently being evaluated for MDS patients with CBL or RAS pathway mutations. The oral MEK inhibitor trametinib has been tested in a phase I/II study for RAS mutant R/R myeloid malignancies, showing modest ORR (20%–27% based on dosing level), but no survival benefit for RAS_mt_ vs RAS_wt_ AML or MDS patients [98]. Rigosertib, a small molecule that induces inhibition of PI3K and PLK pathways [99, 100], showed to have a trend toward improved median OS in MDS patients with HMA failure (8.6 vs. 5.3 months) and post hoc improved survival in high‐risk IPSS‐R patients (7.6 vs. 3.2 months) compared to best supportive care [100]. Finally, pevonedistat, an inhibitor of the NEDD8 (neural precursor cell expressed, developmentally downregulated 8) activating enzyme, has shown superior outcomes in combination with azacitidine, in comparison to the hypomethylating agent alone. In high‐risk MDS patients, median OS (23.9 vs. 19.1 months), EFS (20.2 vs. 14.8 months), ORR (79.3% vs. 56.7%), and CR rate (51.7% vs. 26.7%) all favored the pevonedistat arm [101]. These trials highlight a handful of promising novel agents. Few drugs are currently FDA approved for MDS, and therefore have a unique opportunity to make changes in the field. Recognition that MDS is extremely heterogenous and is impacted by multiple pathways has enhanced our knowledge of the disease but also challenged the progress made to date.

## 10 CONCLUSION

The overwhelming majority of patients with MDS have alterations in genes that affect hematopoietic cell function and likely drive the clinical phenotype of the disease. Our review of the literature details independent prognostic information gained from these mutations, when controlling for other known prognostic variables, which is summarized in Table 1. Mutations in TP53, EZH2, ASXL1, DNMT3A, RUNX1, SRSF2, and CBL have extensive evidence for their negative impact on survival. While these mutations are not currently factored into clinical prognostication models, future consideration when determining patients’ risk and treatment decisions including allotransplant decisions, should be taken into account. It is worth considering that patients with high‐risk genetic features should be evaluated up front for clinical trials with novel single or combination/targeted therapies rather than taking a watch and wait approach. SF3B1 is the lone known mutation carrying favorable prognosis, marking an indolent course defined by transfusion dependence, with a median OS quoted at 7.5 years. On the other hand, data regarding mutations in IDH1/2, TET2, BCOR, ETV6, GATA2, U2AF1, ZRSR2, and RAS pathway and STAG2/cohesin complex suggest an association with neutral or poor prognosis, albeit with a lower amount of evidence, and at times conflicting data. At present, there appear to be insufficient and/or inconsistent data to make definitive conclusions regarding the clinical impact and therapeutic implications for this spectrum of mutations.

Mutational status correlates with specific disease phenotype and outcome and needs to be considered for drug responsiveness and development. For example, SF3B1 mutant patients with ringed sideroblast do very well, while patients with TP53_mut_ disease remains a therapeutic challenge. Table 5 summarizes the current clinical trials that are investigating targeting driver mutations to help improve clinical outcomes for patients with MDS. Based on this review, all patients with confirmed prognostic driver mutations may benefit from investigational targeted agents developed to aid in their survival.

Overall, prospective trials evaluating large cohorts of MDS patients for molecular classification are ongoing by the International Working Group for the prognosis of MDS. These trials will shed light on the net impact of driver mutations on clinical outcomes which should be incorporated into future prognostic scoring systems. In addition, impact of VAFs and class of mutation (frameshift vs. missense mutation vs. deletion vs loss of heterozygosity) for each specific genetic lesion will play a role in the biology of disease and response to treatment, allowing us to distinguish driver versus passenger mutations. We postulate that these data will enhance our prognostic capabilities and our therapeutic decision making to improve patient outcomes.

## CONFLICT OF INTEREST

The authors declare no conflict of interest.

## AUTHOR CONTRIBUTIONS

Michael R. Cook and Catherine Lai designed review concept. Michael R. Cook collected literature and summarized analysis. All the authors wrote and edited the manuscript, and have agreed to the final submitted version.

## References

[1] Ma X , Does M , Raza A , Mayne ST . Myelodysplastic syndromes: incidence and survival in the United States. Cancer 2007;109(8):1536–42. 17345612 10.1002/cncr.22570

[2] Cogle CR , Craig BM , Rollison DE , List AF . Incidence of the myelodysplastic syndromes using a novel claims‐based algorithm: high number of uncaptured cases by cancer registries. Blood 2011;117(26):7121–5. 21531980 10.1182/blood-2011-02-337964 PMC3143554

[3] Swerdlow SH , Campo E , Harris NL , Jaffe ES, Pileri SA, Stein H et al., editors. WHO classification of tumours of haematopoietic and lymphoid tissues, revised 4th edition. Lyon: International Agency for Research on Cancer (IARC); 2017.

[4] Schanz J , Tüchler H , Solé F , Mallo M , Luño E , Cervera J , et al. New comprehensive cytogenetic scoring system for primary myelodysplastic syndromes (MDS) and oligoblastic acute myeloid leukemia after MDS derived from an international database merge. J Clin Oncol. 2012;30(8):820–9. 22331955 10.1200/JCO.2011.35.6394 PMC4874200

[5] Haferlach T , Nagata Y , Grossmann V , Okuno Y , Bacher U , Nagae G , et al. Landscape of genetic lesions in 944 patients with myelodysplastic syndromes. Leukemia 2014;28(2):241–7. 24220272 10.1038/leu.2013.336 PMC3918868

[6] Merino D , Malkin D . p53 and hereditary cancer. Subcell Biochem. 2014;85:1–16. 25201186 10.1007/978-94-017-9211-0_1

[7] Olney HJ, Le Beau MM . Evaluation of recurring cytogenetic abnormalities in the treatment of myelodysplastic syndromes. Leuk Res. 2007;31(4):427–34. 17161457 10.1016/j.leukres.2006.10.023

[8] Christiansen DH , Andersen MK , Pedersen‐Bjergaard J . Mutations with loss of heterozygosity of p53 are common in therapy‐related myelodysplasia and acute myeloid leukemia after exposure to alkylating agents and significantly associated with deletion or loss of 5q, a complex karyotype, and a poor prognosis. J Clin Oncol. 2001;19(5):1405–13. 11230485 10.1200/JCO.2001.19.5.1405

[9] Kaneko H , Misawa S , Horiike S , Nakai H , Kashima K . TP53 mutations emerge at early phase of myelodysplastic syndrome and are associated with complex chromosomal abnormalities. Blood 1995;85(8):2189–93. 7718890

[10] Haase D , Stevenson KE , Neuberg D , Maciejewski JP , Nazha A , Sekeres MA , et al. TP53 mutation status divides myelodysplastic syndromes with complex karyotypes into distinct prognostic subgroups. Leukemia 2019;33(7):1747–58. 30635634 10.1038/s41375-018-0351-2 PMC6609480

[11] Sebaa A , Ades L , Baran‐Marzack F , Mozziconacci MJ , Penther D , Dobbelstein S , et al. Incidence of 17p deletions and TP53 mutation in myelodysplastic syndrome and acute myeloid leukemia with 5q deletion. Genes Chromosomes Cancer. 2012;51(12):1086–92. 22933333 10.1002/gcc.21993

[12] Wattel E , Preudhomme C , Hecquet B , Vanrumbeke M , Quesnel B , Dervite I , et al. p53 mutations are associated with resistance to chemotherapy and short survival in hematologic malignancies. Blood 1994;84(9):3148–57. 7949187

[13] Horiike S , Kita‐Sasai Y , Nakao M , Taniwaki M . Configuration of the TP53 gene as an independent prognostic parameter of myelodysplastic syndrome. Leuk Lymphoma. 2003;44(6):915–22. 12854888 10.1080/1042819031000067620

[14] Kita‐Sasai Y , Horiike S , Misawa S , Kaneko H , Kobayashi M , Nakao M , et al. International prognostic scoring system and TP53 mutations are independent prognostic indicators for patients with myelodysplastic syndrome. Br J Haematol. 2001;115(2):309–12. 11703325 10.1046/j.1365-2141.2001.03073.x

[15] Papaemmanuil E , Gerstung M , Malcovati L , Tauro S , Gundem G , Van Loo P , et al. Clinical and biological implications of driver mutations in myelodysplastic syndromes. Blood 2013;122(22):3616–99. 24030381 10.1182/blood-2013-08-518886 PMC3837510

[16] Bejar R , Stevenson KE , Caughey BA , Abdel‐Wahab O , Steensma DP , Galili N , et al. Validation of a prognostic model and the impact of mutations in patients with lower‐risk myelodysplastic syndromes. J Clin Oncol. 2012;30(27):3376–82. 22869879 10.1200/JCO.2011.40.7379 PMC3438234

[17] Crisà E , Kulasekararaj AG , Adema V , Such E , Schanz J , Haase D , et al. Impact of somatic mutations in myelodysplastic patients with isolated partial or total loss of chromosome 7. Leukemia 2020;34(9):2441–50. 10.1038/s41375-020-0728-x. 32066866

[18] Bejar R , Papaemmanuil E , Haferlach T , Garcia‐Manero G , Maciejewski JP , Sekeres MA , et al. Somatic mutations in MDS patients are associated with clinical features and predict prognosis independent of the IPSS‐R: analysis of combined datasets from the international working group for prognosis in MDS‐molecular committee. Blood 2015;126(23):907.

[19] Bejar R , Stevenson KE , Caughey B , Lindsley RC , Mar BG , Stojanov P , et al. Somatic mutations predict poor outcome in patients with myelodysplastic syndrome after hematopoietic stem‐cell transplantation. J Clin Oncol. 2014;32(25):2691–8. 25092778 10.1200/JCO.2013.52.3381 PMC4207878

[20] Della Porta MG , Gallì A , Bacigalupo A , Zibellini S , Bernardi M , Rizzo E , et al. Clinical effects of driver somatic mutations on the outcomes of patients with myelodysplastic syndromes treated with allogeneic hematopoietic stem‐cell transplantation. J Clin Oncol. 2016;34(30):3627–37. 27601546 10.1200/JCO.2016.67.3616 PMC6366344

[21] Kulasekararaj AG , Smith AE , Mian SA , Mohamedali AM , Krishnamurthy P , Lea NC , et al. TP53 mutations in myelodysplastic syndrome are strongly correlated with aberrations of chromosome 5, and correlate with adverse prognosis. Br J Haematol. 2013;160(5):660–72. 23297687 10.1111/bjh.12203

[22] Hou HA , Tsai CH , Lin CC , Chou WC , Kuo YY , Liu CY , et al. Incorporation of mutations in five genes in the revised International Prognostic Scoring System can improve risk stratification in the patients with myelodysplastic syndrome. Blood Cancer J. 2018;8(4):39. 29618722 10.1038/s41408-018-0074-7 PMC5884776

[23] Bejar R , Stevenson K , Abdel‐Wahab O , Galili N , Nilsson B , Garcia‐Manero G , et al. Clinical effect of point mutations in myelodysplastic syndromes. N Engl J Med. 2011;364(26):2496–506. 21714648 10.1056/NEJMoa1013343 PMC3159042

[24] Xu L , Gu ZH , Li Y , Zhang JL , Chang CK , Pan CM , et al. Genomic landscape of CD34+ hematopoietic cells in myelodysplastic syndrome and gene mutation profiles as prognostic markers. Proc Natl Acad Sci U S A. 2014;111(23):8589–94. 24850867 10.1073/pnas.1407688111 PMC4060725

[25] Montalban‐Bravo G , Pierola AA , Takahashi K , Konopleva M , Jabbour E , Borthakur G , et al. Clinical relevance of mutations in patients with myelodysplastic syndromes and myelodysplastic/myeloproliferative neoplasms with normal karyotype. J Clin Oncol. 2017;35(15_suppl):7053.

[26] Montalban‐Bravo G , Takahashi K , Patel K , Wang F , Xingzhi S , Nogueras GM , et al. Impact of the number of mutations in survival and response outcomes to hypomethylating agents in patients with myelodysplastic syndromes or myelodysplastic/myeloproliferative neoplasms. Oncotarget 2018;9(11):9714–27. 29515765 10.18632/oncotarget.23882 PMC5839396

[27] Al‐Issa K , Zarzour A , Radivoyevitch T , Kalaycio M , Hamilton BK , Gerds AT , et al. Incorporation of molecular data into the current prognostic models in treated patients with myelodysplastic syndromes: which model ss the best. Blood 2016;128(22):50.

[28] Yoshizato T , Nannya Y , Atsuta Y , Shiozawa Y , Iijima‐Yamashita Y , Yoshida K , et al. Genetic abnormalities in myelodysplasia and secondary acute myeloid leukemia: impact on outcome of stem cell transplantation. Blood 2017;129(17):2347–58. 28223278 10.1182/blood-2016-12-754796 PMC5409449

[29] Lindsley RC , Saber W , Mar BG , Redd R , Wang T , Haagenson MD , et al. Prognostic mutations in myelodysplastic syndrome after stem‐cell transplantation. N Engl J Med. 2017;376(6):536–47. 28177873 10.1056/NEJMoa1611604 PMC5438571

[30] Nazha A , Komrokji R , Meggendorfer M , Jia X , Radakovich N , Shreve J , et al. Personalized prediction model to risk stratify patients with myelodysplastic syndromes. J Clin Oncol. 2021. 10.1200/JCO.20.02810 PMC8601291 34406850

[31] Jädersten M , Saft L , Smith A , Kulasekararaj A , Pomplun S , Göhring G , et al. TP53 mutations in low‐risk myelodysplastic syndromes with del(5q) predict disease progression. J Clin Oncol. 2011;29(15):1971–9. 21519010 10.1200/JCO.2010.31.8576

[32] Montalban‐Bravo G , Kanagal‐Shamanna R , Benton CB , Class CA , Chien KS , Sasaki K , et al. Genomic context and TP53 allele frequency define clinical outcomes in TP53‐mutated myelodysplastic syndromes. Blood Adv. 2020;4(3):482–95. 32027746 10.1182/bloodadvances.2019001101 PMC7013259

[33] Bernard E , Nannya Y , Hasserjian RP , Devlin SM , Tuechler H , Medina‐Martinez JS , et al. Implications of TP53 allelic state for genome stability, clinical presentation and outcomes in myelodysplastic syndromes. Nat Med. 2020;26(10):1549–56. 32747829 10.1038/s41591-020-1008-z PMC8381722

[34] Tatton‐Brown K , Rahman N . The NSD1 and EZH2 overgrowth genes, similarities and differences. Am J Med Genet C Semin Med Genet. 2013;163c(2):86–91. 23592277 10.1002/ajmg.c.31359 PMC4845886

[35] Mcgraw KL , Nguyen J , Alali N , Song J , Sallman D , Padron E , et al. EZH2 protein expression is decreased in MDS and MDS/MPN and correlated with EZH2 mutation status, chromosomal 7 abnormalities and clinical outcome. Blood 2016;128(22):4305.

[36] Sakhdari A , Class C , Montalban‐Bravo G , Sasaki K , Bueso‐Ramos CE , Patel KP , et al. Loss of EZH2 protein expression in myelodysplastic syndrome correlates with EZH2 mutation and portends a worse outcome. Blood 2019;134(Supplement_1):3016. 10.1038/s41379-022-01074-y PMC13105204 35504958

[37] Zhang Q , Han Q , Zi J , Ma J , Song H , Tian Y , et al. Mutations in EZH2 are associated with poor prognosis for patients with myeloid neoplasms. Genes Dis. 2019;6(3):276–81. 32042866 10.1016/j.gendis.2019.05.001 PMC6997607

[38] Challen GA , Sun D , Jeong M , Luo M , Jelinek J , Berg JS , et al. Dnmt3a is essential for hematopoietic stem cell differentiation. Nat Genet. 2011;44(1):23–31. 22138693 10.1038/ng.1009 PMC3637952

[39] Lin M‐E , Hou H‐A , Kuo Y‐Y , Chou W‐C , Lee MC , Chen C‐Y , et al. DNMT3A mutations in de novo myelodysplastic syndrome: distinct clinico‐biological features and prognostic relevance. Blood 2012;120(21):3799.

[40] Damm F , Kosmider O , Gelsi‐Boyer V , Renneville A , Carbuccia N , Hidalgo‐Curtis C , et al. Mutations affecting mRNA splicing define distinct clinical phenotypes and correlate with patient outcome in myelodysplastic syndromes. Blood 2012;119(14):3211–8. 22343920 10.1182/blood-2011-12-400994

[41] Lin ME , Hou HA , Tsai CH , Wu SJ , Kuo YY , Tseng MH , et al. Dynamics of DNMT3A mutation and prognostic relevance in patients with primary myelodysplastic syndrome. Clin Epigenetics. 2018;10:42. 29619119 10.1186/s13148-018-0476-1 PMC5879939

[42] Xu Y , Li Y , Xu Q , Chen Y , Lv N , Jing Y , et al. Implications of mutational spectrum in myelodysplastic syndromes based on targeted next‐generation sequencing. Oncotarget 2017;8(47):82475–90. 29137279 10.18632/oncotarget.19628 PMC5669905

[43] Nazha A , Sekeres MA , Bejar R , Rauh MJ , Othus M , Komrokji RS , et al. Genomic biomarkers to predict resistance to hypomethylating agents in patients with myelodysplastic syndromes using artificial intelligence. JCO Precis Oncol. 2019;3. 10.1200/po.19.00119. PMC6818517 31663066

[44] Walter MJ , Ding L , Shen D , Shao J , Grillot M , McLellan M , et al. Recurrent DNMT3A mutations in patients with myelodysplastic syndromes. Leukemia 2011;25(7):1153–8. 21415852 10.1038/leu.2011.44 PMC3202965

[45] Liang S , Zhou X , Pan H , Yang Y , Shi L , Wang L . Prognostic value of DNMT3A mutations in myelodysplastic syndromes: a meta‐analysis. Hematology 2019;24(1):613–22. 31482762 10.1080/16078454.2019.1657613

[46] Inoue D , Kitaura J , Togami K , Nishimura K , Enomoto Y , Uchida T , et al. Myelodysplastic syndromes are induced by histone methylation–altering ASXL1 mutations. J Clin Invest. 2013;123(11):4627–40. 24216483 10.1172/JCI70739 PMC3809801

[47] Thol F , Friesen I , Damm F , Yun H , Weissinger EM , Krauter J , et al. Prognostic significance of ASXL1 mutations in patients with myelodysplastic syndromes. J Clin Oncol. 2011;29(18):2499–506. 21576631 10.1200/JCO.2010.33.4938

[48] Tefferi A , Gangat N , Mudireddy M , Lasho TL , Finke C , Begna KH , et al. Mayo alliance prognostic model for myelodysplastic syndromes: integration of genetic and clinical information. Mayo Clin Proc. 2018;93(10):1363–74. 29866419 10.1016/j.mayocp.2018.04.013

[49] Gangat N , Mudireddy M , Lasho TL , Finke CM , Nicolosi M , Szuber N , et al. Mutations and prognosis in myelodysplastic syndromes: karyotype‐adjusted analysis of targeted sequencing in 300 consecutive cases and development of a genetic risk model. Am J Hematol. 2018;93(5):691–7. 29417633 10.1002/ajh.25064

[50] Bacher U , Haferlach T , Schnittger S , Zenger M , Meggendorfer M , Jeromin S , et al. Investigation of 305 patients with myelodysplastic syndromes and 20q deletion for associated cytogenetic and molecular genetic lesions and their prognostic impact. Br J Haematol. 2014;164(6):822–33. 24372512 10.1111/bjh.12710

[51] Losman J‐A , Looper RE , Koivunen P , Lee S , Schneider RK , McMahon C , et al. (R)‐2‐hydroxyglutarate is sufficient to promote leukemogenesis and its effects are reversible. Science 2013;339(6127):1621–5. 23393090 10.1126/science.1231677 PMC3836459

[52] Thol F , Weissinger EM , Krauter J , Wagner K , Damm F , Wichmann M , et al. IDH1 mutations in patients with myelodysplastic syndromes are associated with an unfavorable prognosis. Haematologica 2010;95(10):1668–74. 20494930 10.3324/haematol.2010.025494 PMC2948091

[53] Patnaik MM , Hanson CA , Hodnefield JM , Lasho TL , Finke CM , Knudson RA , et al. Differential prognostic effect of IDH1 versus IDH2 mutations in myelodysplastic syndromes: a Mayo Clinic study of 277 patients. Leukemia 2012;26(1):101–5. 22033490 10.1038/leu.2011.298

[54] Wang N , Wang F , Shan N , Sui X , Xu H . IDH1 mutation is an independent inferior prognostic indicator for patients with myelodysplastic syndromes. Acta Haematol. 2017;138(3):143–51. 28873367 10.1159/000479546

[55] Lin CC , Hou HA , Chou WC , Kuo YY , Liu CY , Chen CY , et al. IDH mutations are closely associated with mutations of DNMT3A, ASXL1 and SRSF2 in patients with myelodysplastic syndromes and are stable during disease evolution. Am J Hematol. 2014;89(2):137–44. 24115220 10.1002/ajh.23596

[56] Jin J , Hu C , Yu M , Chen F , Ye L , Yin X , et al. Prognostic value of isocitrate dehydrogenase mutations in myelodysplastic syndromes: a retrospective cohort study and meta‐analysis. PLoS One. 2014;9(6):e100206. 24936872 10.1371/journal.pone.0100206 PMC4061070

[57] DiNardo CD , Jabbour E , Ravandi F , Takahashi K , Daver N , Routbort M , et al. IDH1 and IDH2 mutations in myelodysplastic syndromes and role in disease progression. Leukemia 2016;30(4):980–4. 26228814 10.1038/leu.2015.211 PMC4733599

[58] Delhommeau F , Dupont S , James C , Masse A , le Couedic JP , Valle VD , et al. TET2 Is a novel tumor suppressor gene inactivated in myeloproliferative neoplasms: identification of a pre‐JAK2 V617F event. Blood 2008;112(11). 10.1182/blood.V112.11.lba-3.lba-3

[59] Kosmider O , Gelsi‐Boyer V , Cheok M , Grabar S , Della‐Valle V , Picard F , et al. TET2 mutation is an independent favorable prognostic factor in myelodysplastic syndromes (MDSs). Blood 2009;114(15):3285–91. 19666869 10.1182/blood-2009-04-215814

[60] Smith AE , Mohamedali AM , Kulasekararaj A , Lim Z , Gäken J , Lea NC , et al. Next‐generation sequencing of the TET2 gene in 355 MDS and CMML patients reveals low‐abundance mutant clones with early origins, but indicates no definite prognostic value. Blood 2010;116(19):3923‐32. 20693430 10.1182/blood-2010-03-274704

[61] Guo Z , Zhang SK , Zou Z , Fan RH , Lyu XD . Prognostic significance of TET2 mutations in myelodysplastic syndromes: a meta‐analysis. Leuk Res. 2017;58:102–7. 28521175 10.1016/j.leukres.2017.03.013

[62] Jiang L , Luo Y , Zhu S , Wang L , Ma L , Zhang H , et al. Mutation status and burden can improve prognostic prediction of patients with lower‐risk myelodysplastic syndromes. Cancer Sci. 2020;111(2):580–91. 31804030 10.1111/cas.14270 PMC7004535

[63] Cao Q , Gearhart MD , Gery S , Shojaee S , Yang H , Sun H , et al. BCOR regulates myeloid cell proliferation and differentiation. Leukemia 2016;30(5):1155–65. 26847029 10.1038/leu.2016.2 PMC5131645

[64] Damm F , Chesnais V , Nagata Y , Yoshida K , Scourzic L , Okuno Y , et al. BCOR and BCORL1 mutations in myelodysplastic syndromes and related disorders. Blood 2013;122(18):3169–77. 24047651 10.1182/blood-2012-11-469619

[65] Abuhadra N , Al‐Issa K , Mukherjee S , Hirsch CM , Gerds AT , Jha BK , et al. BCOR mutations in myelodysplastic syndromes (MDS): mutation characteristics impact clinical outcomes. Blood 2017;130(Supplement 1):5304.

[66] Lam K , Zhang DE . RUNX1 and RUNX1‐ETO: roles in hematopoiesis and leukemogenesis. Front Biosci. 2012;17:1120–39. 10.2741/3977 PMC3433167 22201794

[67] Osato M . Point mutations in the RUNX1/AML1 gene: another actor in RUNX leukemia. Oncogene 2004;23(24):4284–96. 15156185 10.1038/sj.onc.1207779

[68] Chen CY , Lin LI , Tang JL , Ko BS , Tsay W , Chou WC , et al. RUNX1 gene mutation in primary myelodysplastic syndrome–the mutation can be detected early at diagnosis or acquired during disease progression and is associated with poor outcome. Br J Haematol. 2007;139(3):405–14. 17910630 10.1111/j.1365-2141.2007.06811.x

[69] Wang LC , Swat W , Fujiwara Y , Davidson L , Visvader J , Kuo F , et al. The TEL/ETV6 gene is required specifically for hematopoiesis in the bone marrow. Genes Dev. 1998;12(15):2392–402. 9694803 10.1101/gad.12.15.2392 PMC317042

[70] Crispino JD . GATA1 in normal and malignant hematopoiesis. Semin Cell Dev Biol. 2005;16(1):137–47. 15659348 10.1016/j.semcdb.2004.11.002

[71] Dickinson RE , Milne P , Jardine L , Zandi S , Swierczek SI , McGovern N , et al. The evolution of cellular deficiency in GATA2 mutation. Blood 2014;123(6):863–74. 24345756 10.1182/blood-2013-07-517151 PMC3916878

[72] Papaemmanuil E , Cazzola M , Boultwood J , Malcovati L , Vyas P , Bowen D , et al. Somatic SF3B1 mutation in myelodysplasia with ring sideroblasts. N Engl J Med. 2011;365(15):1384–95. 21995386 10.1056/NEJMoa1103283 PMC3322589

[73] Yoshida K , Sanada M , Shiraishi Y , Nowak D , Nagata Y , Yamamoto R , et al. Frequent pathway mutations of splicing machinery in myelodysplasia. Nature 2011;478(7367):64–9. 21909114 10.1038/nature10496

[74] Malcovati L , Stevenson K , Papaemmanuil E , Neuberg D , Bejar R , Boultwood J , et al. SF3B1‐mutant MDS as a distinct disease subtype: a proposal from the International Working Group for the Prognosis of MDS. Blood 2020;136(2):157–70. 32347921 10.1182/blood.2020004850 PMC7362582

[75] Malcovati L , Papaemmanuil E , Bowen DT , Boultwood J , Della Porta MG , Pascutto C , et al. Clinical significance of SF3B1 mutations in myelodysplastic syndromes and myelodysplastic/myeloproliferative neoplasms. Blood 2011;118(24):6239–46. 21998214 10.1182/blood-2011-09-377275 PMC3236114

[76] Long JC , Caceres JF . The SR protein family of splicing factors: master regulators of gene expression. Biochem J. 2009;417(1):15–27. 19061484 10.1042/BJ20081501

[77] Thol F , Kade S , Schlarmann C , Löffeld P , Morgan M , Krauter J , et al. Frequency and prognostic impact of mutations in SRSF2, U2AF1, and ZRSR2 in patients with myelodysplastic syndromes. Blood 2012;119(15):3578–84. 22389253 10.1182/blood-2011-12-399337

[78] Arbab Jafari P , Ayatollahi H , Sadeghi R , Sheikhi M , Asghari A . Prognostic significance of SRSF2 mutations in myelodysplastic syndromes and chronic myelomonocytic leukemia: a meta‐analysis. Hematology 2018;23(10):778–84. 29757120 10.1080/10245332.2018.1471794

[79] Graubert TA , Shen D , Ding L , Okeyo‐Owuor T , Lunn CL , Shao J , et al. Recurrent mutations in the U2AF1 splicing factor in myelodysplastic syndromes. Nat Genet. 2011;44(1):53–7. 22158538 10.1038/ng.1031 PMC3247063

[80] Nadeau S , An W , Palermo N , Feng D , Ahmad G , Dong L , et al. Oncogenic signaling by leukemia‐associated mutant Cbl proteins. Biochem Anal Biochem. 2012;1(Suppl 6):7921. 10.4172/2161-1009.S6-001 PMC3757940 23997989

[81] Sanada M , Suzuki T , Shih LY , Otsu M , Kato M , Yamazaki S , et al. Gain‐of‐function of mutated C‐CBL tumour suppressor in myeloid neoplasms. Nature 2009;460(7257):904–8. 19620960 10.1038/nature08240

[82] Hancock JF . Ras proteins: different signals from different locations. Nat Rev Mol Cell Biol. 2003;4(5):373–84. 12728271 10.1038/nrm1105

[83] Barbero JL . Cohesins: chromatin architects in chromosome segregation, control of gene expression and much more. Cell Mol Life Sci. 2009;66(13):2025–35. 19290475 10.1007/s00018-009-0004-8 PMC11115881

[84] Thota S , Viny AD , Makishima H , Spitzer B , Radivoyevitch T , Przychodzen B , et al. Genetic alterations of the cohesin complex genes in myeloid malignancies. Blood 2014;124(11):1790–8. 25006131 10.1182/blood-2014-04-567057 PMC4162108

[85] Strom SP . Current practices and guidelines for clinical next‐generation sequencing oncology testing. Cancer Biol Med. 2016;13(1):3–11. 27144058 10.28092/j.issn.2095-3941.2016.0004 PMC4850126

[86] Greenberg PL , Tuechler H , Schanz J , Sanz G , Garcia‐Manero G , Solé F , et al. Revised international prognostic scoring system for myelodysplastic syndromes. Blood 2012;120(12):2454–65. 22740453 10.1182/blood-2012-03-420489 PMC4425443

[87] Greenberg P , Tuechler H , Schanz J , Sole F , Bennett J , Garcia‐Manero G , et al. 14 revised International Prognostic Scoring System (IPSS‐R), developed by the International Prognostic Working Group for Prognosis in MDS (IWG‐PM). Leuk Res. 2011;35:S6.

[88] Duncavage EJ , Schroeder MC , O'Laughlin M , Wilson R , MacMillan S , Bohannon A , et al. Genome sequencing as an alternative to cytogenetic analysis in myeloid cancers. N Engl J Med. 2021;384(10):924–35. 33704937 10.1056/NEJMoa2024534 PMC8130455

[89] Itzykson R , Fournier E , Berthon C , Röllig C , Braun T , Marceau‐Renaut A , et al. Genetic identification of patients with AML older than 60 years achieving long‐term survival with intensive chemotherapy. Blood 2021;138(7):507–19. 34410352 10.1182/blood.2021011103

[90] Zhou L , Nguyen AN , Sohal D , Ying Ma J , Pahanish P , Gundabolu K , et al. Inhibition of the TGF‐beta receptor I kinase promotes hematopoiesis in MDS. Blood 2008;112(8):3434–43. 18474728 10.1182/blood-2008-02-139824 PMC2569182

[91] Fenaux P , Platzbecker U , Mufti GJ , Garcia‐Manero G , Buckstein R , Santini V , et al. Luspatercept in patients with lower‐risk myelodysplastic syndromes. N Engl J Med. 2020;382(2):140–51. 31914241 10.1056/NEJMoa1908892

[92] Sallman DA , DeZern AE , Garcia‐Manero G , Steensma DP , Roboz GJ , Sekeres MA , et al. Phase 2 results of APR‐246 and azacitidine (AZA) in patients with TP53 mutant myelodysplastic syndromes (MDS) and oligoblastic acute myeloid leukemia (AML). Blood 2019;134(Supplement_1):676.

[93] Coiante MS , Korbel AG , Attar E . Aprea therapeutics announces results of primary endpoint from Phase 3 trial of eprenetapopt in TP53 mutant myelodysplastic syndromes (MDS). aprea therapeutics; 2020.

[94] Sallman DA , Malki MA , Asch AS , Lee DJ , Kambhampati S , Donnellan WB , et al. Tolerability and efficacy of the first‐in‐class anti‐CD47 antibody magrolimab combined with azacitidine in MDS and AML patients: phase Ib results. J Clin Oncol. 2020;38(15_suppl):7507.

[95] Watts J , Baer MR , Yang J , Dinner S , Lee S , Seiter K , et al. Phase 1 study of the IDH1m inhibitor FT‐2102 as a single agent in patients with IDH1m acute myeloid leukemia (AML) or myelodysplastic syndrome (MDS). Blood 2018;132(Supplement 1):1453.

[96] Cortes JE , Watts J , Prebet T , Schiller GJ , Lee S , Yang J , et al. FT‐2102, an IDH1m inhibitor, in combination with azacitidine in patients with acute myeloid leukemia (AML) or myelodysplastic ayndrome (MDS): results from a Phase 1 study. Blood 2018;132(Supplement 1):1452.30097508

[97] DiNardo CD , Foran JM , Watts JM , Stein EM , de Botton S , Fathi AT , et al. Ivosidenib (IVO; AG‐120) in IDH1‐mutant relapsed or refractory myelodysplastic syndrome: updated results from a phase 1 study. Clin Lymphoma Myelom Leukemia. 2019;19:S340.

[98] Borthakur G , Popplewell L , Boyiadzis M , Foran J , Platzbecker U , Vey N , et al. Activity of the oral mitogen‐activated protein kinase kinase inhibitor trametinib in RAS‐mutant relapsed or refractory myeloid malignancies. Cancer 2016;122(12):1871–9. 26990290 10.1002/cncr.29986 PMC5779863

[99] Aleshin A , Greenberg PL . Molecular pathophysiology of the myelodysplastic syndromes: insights for targeted therapy. Blood Adv. 2018;2(20):2787–97. 30352953 10.1182/bloodadvances.2018015834 PMC6199665

[100] Garcia‐Manero G , Fenaux P , Al‐Kali A , Baer MR , Sekeres MA , Roboz GJ , et al. Rigosertib versus best supportive care for patients with high‐risk myelodysplastic syndromes after failure of hypomethylating drugs (ONTIME): a randomised, controlled, phase 3 trial. Lancet Oncol. 2016;17(4):496–508. 26968357 10.1016/S1470-2045(16)00009-7

[101] Sekeres MA , Watts J , Radinoff A , Sangerman MA , Cerrano M , Lopez PF , et al. Randomized phase 2 trial of pevonedistat plus azacitidine versus azacitidine for higher‐risk MDS/CMML or low‐blast AML. Leukemia 2021;35(7):2119–24. 33483617 10.1038/s41375-021-01125-4 PMC8257476

